# An Immune Checkpoint-Related Gene Signature for Predicting Survival of Pediatric Acute Myeloid Leukemia

**DOI:** 10.1155/2021/5550116

**Published:** 2021-04-19

**Authors:** Feng Jiang, Xin-Yu Wang, Ming-Yan Wang, Yan Mao, Xiao-Lin Miao, Chu-Yan Wu, Guo-Ping Zhou

**Affiliations:** ^1^Department of Pediatrics, The First Affiliated Hospital of Nanjing Medical University, Nanjing 210000, China; ^2^Neonatal Department, Obstetrics and Gynecology Hospital of Fudan University, Shanghai 200011, China; ^3^Department of Rehabilitation Medicine, The First Affiliated Hospital of Nanjing Medical University, Nanjing 210000, China

## Abstract

**Objective:**

The aim of this research was to create a new genetic signature of immune checkpoint-associated genes as a prognostic method for pediatric acute myeloid leukemia (AML).

**Methods:**

Transcriptome profiles and clinical follow-up details were obtained in Therapeutically Applicable Research to Generate Effective Treatments (TARGET), a database of pediatric tumors. Secondary data was collected from the Gene Expression Omnibus (GEO) to test the observations. In univariate Cox regression and multivariate Cox regression studies, the expression of immune checkpoint-related genes was studied. A three-mRNA signature was developed for predicting pediatric AML patient survival. Furthermore, the GEO cohort was used to confirm the reliability. A bioinformatics method was utilized to identify the diagnostic and prognostic value.

**Results:**

A three-gene (STAT1, BATF, EML4) signature was developed to identify patients into two danger categories depending on their OS. A multivariate regression study showed that the immune checkpoint-related signature (STAT1, BATF, EML4) was an independent indicator of pediatric AML. By immune cell subtypes analyses, the signature was correlated with multiple subtypes of immune cells.

**Conclusion:**

In summary, our three-gene signature can be a useful tool to predict the OS in AML patients.

## 1. Introduction

Acute myeloid leukemia (AML) in children is a progressive disorder with a poor prognosis [1]. In recent decades, the overall survival (OS) of pediatric patients with AML has increased. Since the introduction of high-dose cytarabine/mitoxantrone, the 5-year probability of OS rose significantly from 49% to 76%, but the probability of event-free survival only increased from 41% to 50% and has stayed steady since then, according to a retrospective review of 1940 pediatric AML patients. Despite the increased first-line therapy, non-response and relapse rates remained stable [2]. Another large cohort study involving 482 children with AML showed that significant improvements in patient stratification and optimization in induction and postremission treatment strategies led to an increase in OS [3]. We now have a greater understanding of the specific biology driving pediatric AML and patient results due to decades of concerted efforts across cooperative community trials. The two standard therapies for AML are chemotherapy and hematopoietic stem cell transplantation (HSCT). The 5-year survival rate is still below 50% [4, 5]. Acute promyelocytic leukemia (APL), also known as M3 in the French-American-British system, is a form of AML that affects between 5 and 10% of children in the United States. In today's traditional frontline therapy for pediatric APL, all-trans retinoic acid (ATRA) is included in any step of treatment, resulting in a 90–95 percent full remission rate [6]. Chemotherapy and hematopoietic stem cell transplantation (HSCT) are not even needed any longer [6]. Despite advances in diagnostic methods and therapeutic effectiveness for AML, refractory acute leukemia still reacts and dies during remission, with limited survival duration. Despite the detection of various genetic alterations, including MLL gene rearrangements, Annalisa reported that the histone methyltransferase DOT1L was active in the proliferation of MLL-r cells, for which a target inhibitor, Pinometostat, has been tested in a clinical trial involving pediatric MLL-r leukemic patients [7]. Elena found that exposing MLL-AF6-rearranged AML blasts to tipifarnib, a RAS inhibitor, causes cell autophagy and apoptosis, implying that RAS targeting may be a new therapeutic approach for patients with T cell lymphoma (6; 11) [8]. There are still few more targeted interventions that are effective. As a consequence, early prognostic indications and new therapeutic targets are in high demand.

With the recent advances in microarray technology and bioinformatics, the complex molecular structure of AML has permitted the classification, prognostic stratification, and the discovery of novel drug targets [9]. A risk classification model was proposed by Ng et al. [10], which is focused on 17 gene expression for rapid screening in patients with acute leukemia, and a model of somatic mutations was proposed by Patel et al. [11], which is based on molecular biology of a collection of 18 genes. These models were shown to be predictive of patient outcomes.

The advent of immune checkpoint inhibitors has enabled the treatment of patients with tumors and with a substantial benefit. The immune system's functions during cancer development are complex. When the immune system recognizes tumor cell antigens, it activates both the innate and adaptive immune systems, which are both involved in a range of immune cells and cytokines [12]. However, cancer can cause immune system dysfunction during tumorigenesis and development. The immune system can then become an accomplice through chronic inflammation [13]. Avoiding immune destruction and tumor-promoting inflammation are two hallmarks of cancer immunity [14]. There are several malignancies that need PD-L1/CTLA-4 inhibitors, while other molecules that interrupt inhibitory mechanisms are being studied [15]. Interest in checkpoint inhibitors in AML is currently growing since they help improve the immune response to tumor cells [16]. This specific technique has been used in adult solid cancers but remains unproven in AML. Active therapies available include nivolumab, pembrolizumab, and ipilimumab, which target either CTLA-4, PD-1, or both. Clinical trials have demonstrated that checkpoint inhibition, either alone or in combination with other therapies, is a feasible and effective approach for AML and is currently being explored in large studies [17, 18]. Nivolumab has also proved useful in patients who relapse after stem cell transplant and is an important alternative in this patient group [19]. The only recorded instance of usage of pediatric AML was in a child who had relapsed AML who failed to provide any substantial progress, though step I and II clinical trials in children with AML have been performed [20]. There is a need for appropriate biomarkers for children with AML that can enhance survival estimation and diagnosis. Therefore, focused on immune checkpoint and diagnostic genes, we are utilizing two datasets to confirm a predictive signature for pediatric AML and contribute to assessing successful immunotherapy for pediatric AML.

## 2. Materials and Methods

### 2.1. Immune Checkpoint-Related Gene Collection and Data Acquisition

A total of 187 AML mRNA data and subsequent clinical follow-ups were downloaded from the database of Therapeutically Applicable Research to Generate Effective Treatments (TARGET). The signature was validated using RNA-sequencing data from 417 patients with corresponding clinical follow-ups retrieved from Gene Expression Omnibus (GSE37642). Genes for PD-1/PD-L1 and CTLA-4 signaling pathways were obtained from the KEGG (Kyoto Encyclopedia of Genes and Genomes) and Reactome. The KEGG and Reactome pathway databases resulted in 282 candidate genes (Table S1). For the following study, intersection combinations between immune checkpoint-related genes and the two datasets (TARGET and GSE37642) were analyzed.

### 2.2. Identification of Predictive Genes and Construction of Gene Signature

With a univariate Cox regression study, the connection between genes correlated with immune checkpoint and overall survival (OS) in AML was determined. For the LASSO-penalized Cox regression study by 10-time cross-validation using “glmnet” R package, the gene scale was further reduced in a univariate analysis by *p* < 0.05. Finally, a multivariate approach was used to classify the ideal model with the lowest Akaike data criterion, which is a fitness indicator [21]. The gene-dependent prognosis risk score was calculated based on the defined immune checkpoint and a linear combination of risk score formula and multiple regression degree expression (*β*). Risk score = *β*_1_^∗^ gene_1_expression + *β*_2_^∗^ gene_2_expression + *β*_3_^∗^ gene_3_expression + … + *β*_n_^∗^ gene_n_expression. Risk score was then obtained depending on each patient's algorithm. The median value of the risk score was used as a cutoff in all individuals categorized into high-risk and low-risk categories. In order to compare statistical variance between high- and low-risk categories, Kaplan–Meier study was conducted. Area under the curve (AUC) for 1-, 3-, and 5-year OS was carried out for a time-dependent ROC (receiver operating characteristic) curve to assess the clinically predictive capability of the model.

### 2.3. Prognostic Signature Independence

The study of univariate Cox regression was done in order to assess the significance of current gene signature and clinical parameters on the method of OS in children with AML. Further study of the multivariate Cox regression to classify individual prognostic variables was performed. Survival evaluation was performed to verify the novel signature's risk stratification capability as patients were identified as clinical subgroups.

### 2.4. Gene Set Enrichment Analysis

In order to research the biological mechanisms behind the predictive signature, GSEA investigates whether there are statistically meaningful variations between high- and low-risk classes in defined sets of genes [22]. Sets of genes *p* < 0.05 and FDR < 0.25 were deemed substantially enriched and biological mechanisms were established.

### 2.5. Immune Cell Subtypes and Associations of Immune Checkpoint-Related Genes Defined

The analytic method named CIBERSORT was applied to measure immune cell subtypes in order to examine the relative tumor-infiltrating immune cells' abundance from gene expression profiles in AML. The algorithm calculated the supposed immune cells' abundance by using a comparison range of 1000 permutations of 22 immune cell subtypes (LM22) [23]. In order to determine immune violations of each sample, we used the mRNA expressions matrix as input files [24]. CIBERSORT production of *p* < 0.05 was filtered for subsequent study, indicating inferred proportion of CIBERSORT generated immune cell number is precise [25]. The performance values of the CIBERSORT have been identified as fraction immune cell subtypes. The sum of 22 immune cell fractions for each event was equivalent to 1. Spearman's rank correlation study was conducted on the R-software and the correlations of function genes with infiltrating immune cells were visualized using a kit of “ggplot2.”

### 2.6. Statistical Analysis

Using the package “survival,” survival curves were created. The R package “survivalROC” was used to execute the ROC curves. Multivariate Cox 95 percent confidence interval (CI) relative hazards regression analyses were introduced to classify prospective factors. The visualization was achieved by using the “corrplot” package R of 22 types of infiltrating immune cells. *p* < 0.05 has been found meaningful. Both analyses of statistics were completed with R (version 3.6.2).

### 2.7. PPI Network

Cytoscape is an open source framework for the simulation and integration of dynamic networks with attribute data of any type. Cytoscape has been used for creating a network of protein interactions and evaluating the interaction of core genes in immune control genes. The Cytoscape Network Analyzer was applied for the node degree measurement specified as the number of connections to select key genes in the PPI.

## 3. Results

### 3.1. Clinical Information and Patient Demographics

**Figure 1:**
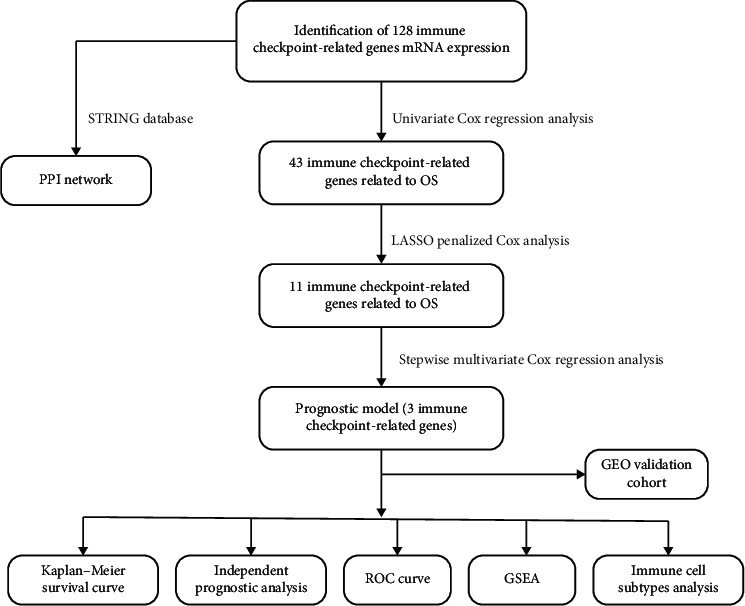
Analysis process flowchart in this study.

The TARGET and GEO cohort clinicopathology features are listed in Table 1. In this study, the survival analysis included clinicopathology and follow-up details composed of 187 AML children in TARGET database and 422 AML patients in GEO database, respectively.  Figure 1 shows the workflow chart.

### 3.2. Gene Feature Identification and Construction of Predictive Gene Signature

**Figure 2:**
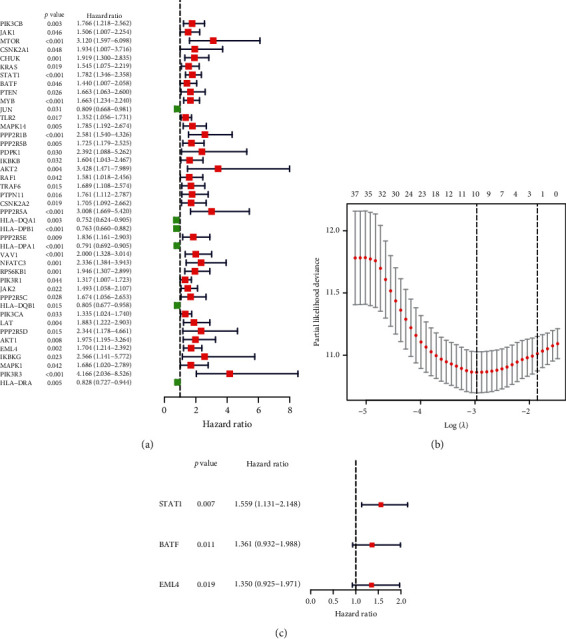
Participant immune checkpoint genes recognition of the TARGET cohort. (a) Univariate Cox regression testing that defines HR prediction variables with 95% CI and *p* values. (b) Selecting LASSO regression algorithm input variables. (c) Constructing immune checkpoint-related gene model by multivariate Cox regression in TARGET cohort.

For subsequent study, a total of 128 genes linked to immune checkpoint between two datasets were established. We also performed PPI network analysis using STRING online tool and Cytoscape software to better clarify associations between these genes that are linked to immune checkpoint (Figure S1). Word clouds are seen in Figure S1 for the 128 immune checkpoint-related genes. Univariate Cox study of 43 survival-related genes and 11 genes preserved after LASSO Cox regression was established (Figures 2(a)–2(b)). Then, multivariate Cox regression study was performed to establish the risk signature (Figure 2(c)). Thus, STAT1, BATF, and EML4 were considerably determined to be key genes linked to prognosis. For each sample, the risk score value for each sample was determined as follows: risk score = 0.4439 ^∗^ STAT1 expression + 0.3082 ^∗^ BATF expression + 0.3003 ^∗^ EML4 expression. Three key genes were all high-risk genes and correlated with poor survival. The risk score in the TARGET and GEO datasets was measured for each person and patients were divided into low- or high-risk categories.

### 3.3. Gene Signature's Performance

**Figure 3:**
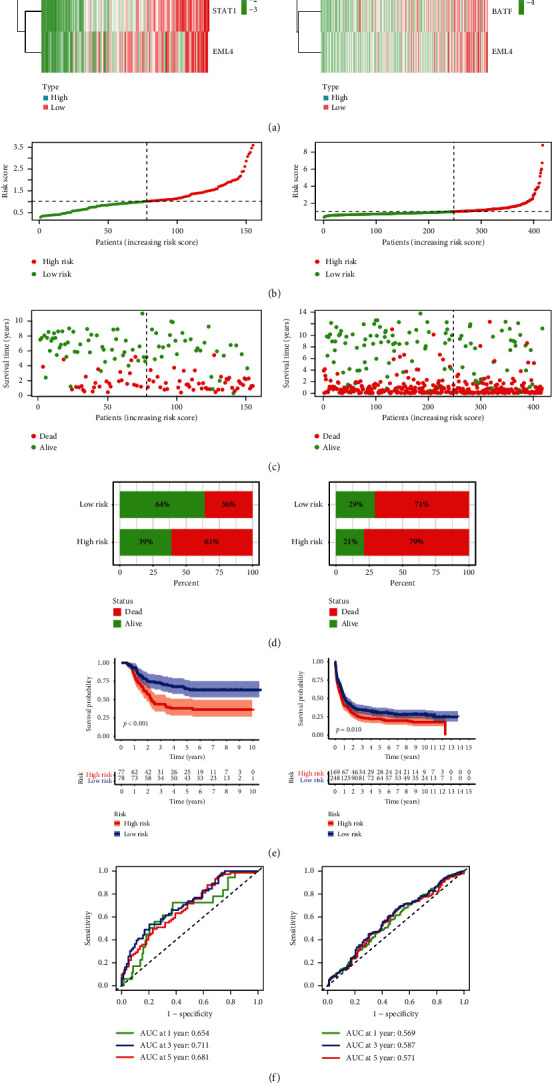
Signature prediction value in pediatric AML. (a) A heat map showing the three patterns of gene expression linked to immune checkpoint in both the TARGET and GEO categories of high and low risk. (b) The distribution of risk score. (c) Distribution of status in high- and low-risk AML patients. The dot indicates the condition of the patient by the rising risk. The *x*-axis consists of the number of patients with a *y*-axis of time of survival. (d) The death rates of all the risk categories. (e), (f) The general survival curves of Kaplan–Meier patients allocated to high- and low-risk groups depending on the median score of the risk score.

The high-risk group AML patients exhibited considerably unfavorable OS in the TARGET cohort (Figure 3(e)) and were further confirmed in GEO dataset relative to low-risk group. The prediction signature AUC values were 0.654, 0.711, and 0.681, respectively, in TARGET dataset for 1-, 3-, and 5-year survival rates (Figure 3(f)). Figures 3(b) and 3(c) show the expression of gene signature between 2 datasets, the risk score distribution, and the survival status of all patients. The prognostic signature can divide patients with AML into low- or high-risk categories, and patients in the TARGET cohort have an elevated risk score, which improves the expression of prognostic genes. In comparison, GEO cohort AUC values were 1, 3, and 5 years, respectively, of 0.569, 0.587, and 0.571 (Figure 3(f)). The higher mortality rate was correlated with an improved risk score (Figure 3(d)). These observations verified the specific prediction of AML patients by the current signature.

### 3.4. Independent Immune Checkpoint-Related Predictor Value

The analysis for multivariate Cox regression of clinicopathological variables was first conducted to decide whether the risk score in the TARGET cohort was an independent predictor of OS. After adjustment for other explanatory factors, it was indicated that the risk score was significantly related to the OS of AML patients (Table 2).

### 3.5. Gene Set Enrichment Analyses

**Figure 4:**
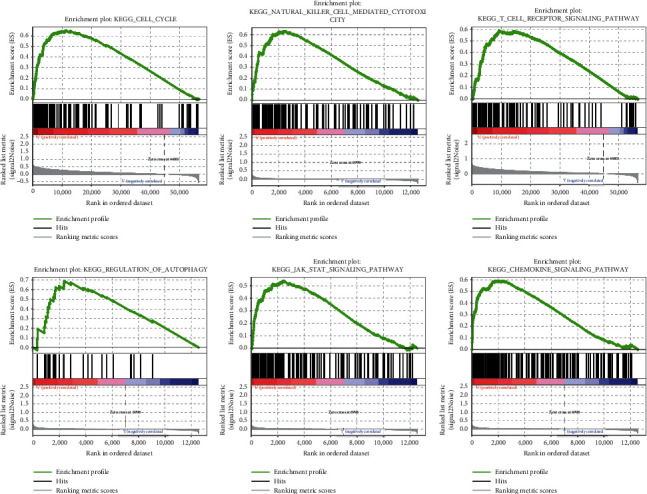
Low-risk and high-risk GSEA enrichment.

GSEA was performed to demonstrate biological pathways between high- and low-risk categories. The biological pathways in high-risk group were significantly enriched by the natural killer cytotoxicity, the T cell receptor signaling pathway, regulation of autophagy, JAK STAT signaling pathway, chemokine signaling pathway, and cell cycle (Figure 4).

### 3.6. Infiltration of Immune Cells and Interaction of Three Immune Checkpoint-Related Genes

**Figure 5:**
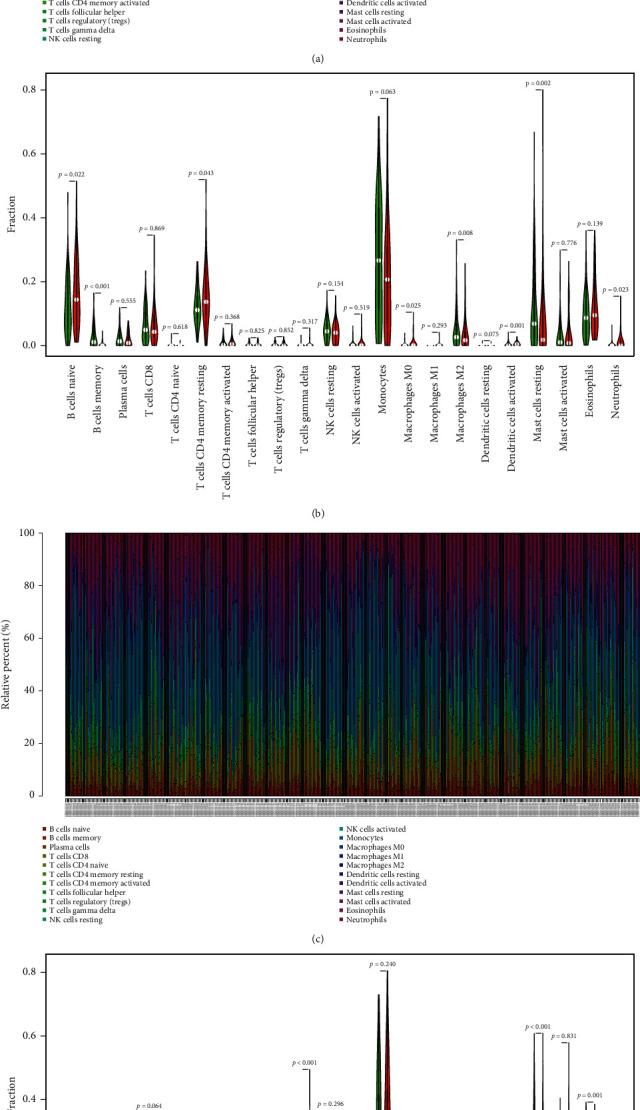
Immune cell subtypes distribution and visualization of patients with AML. (a) Overview of 22 immune cell subtypes in dataset of TARGET predicted compositions. (b) Distinctions between low- and high-risk groups with 22 immune cell subtypes. (c) Description of the 22 immune cell subtypes of the GEO cohort estimated compositions. (d) Contrast between low- and high-risk groups in 22 subtypes of immune cells. Colors blue and red represent low-risk and high-risk samples.

**Figure 6:**
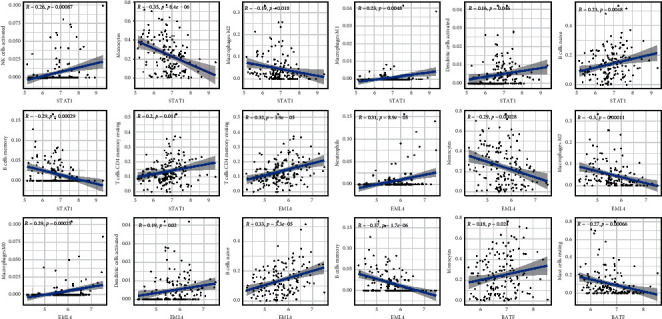
STAT1, BATF, and EML4 association and infiltration of immune cells in pediatric AML.

In AML patients from the TARGET (Figure 5(a)) and GEO cohorts, we first examined immune cell composition (Figure 5(c)). TARGET cohort has considerably higher proportions of naive, T cell CD4 memory in high-risk group than in low-risk group (Figure 5(b)). The mast cells and macrophages M2 proportion were therefore significantly less than in low-risk populations. The GEO cohort was higher than low-risk group in proportions of naive B cells and T-gamma delta cells. However, the proportion of resting mast cells and eosinophils in the low-risk group is relatively lower. As Figure 6 shows, STAT1 has been positively associated with activated NK cells, macrophages M1, and activated dendritic cells and negatively associated with monocytes, macrophages M2, and memory B cells. T CD4 memory resting, neutrophils, macrophages M0, dendritic cell activated, and naive B cell were positively correlated with EML4 and negatively associated with monocytes, macrophages M2, and B cell memory. BATF was linked to monocytes with memory resting T cells CD4 and to mast cells resting negatively.

### 3.7. Risk Score Ability as an Indicator of Immunotherapy Response

**Figure 7:**
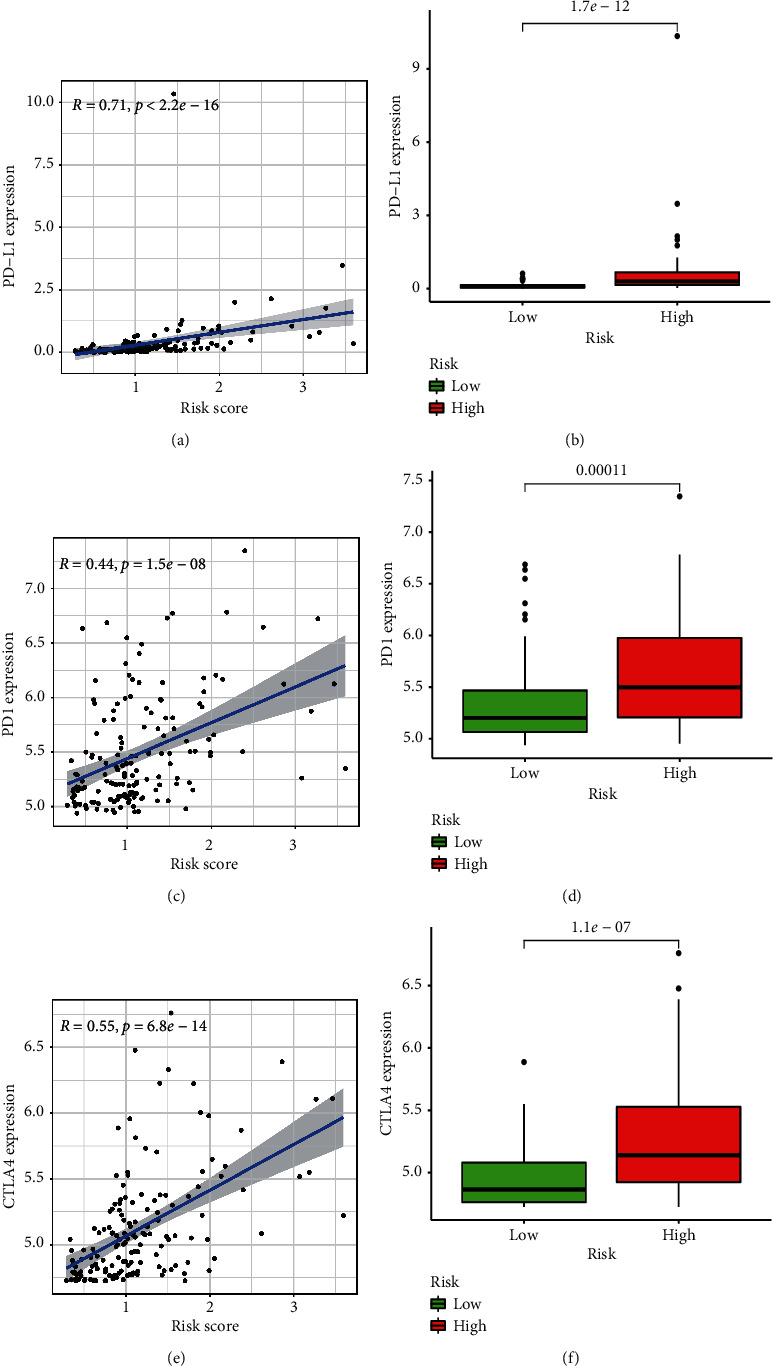
Comparison of risk score and three immune checkpoint gene expressions in TARGET cohort. (a) Correlation between PD-L1 expression and risk score. (b) PD-L1 expression in high and low-risk group. (c) Correlation between PD1 expression and risk score. (d) PD1 expression in high- and low-risk group. (e) Correlation between CTLA4 expression and risk score. (f) CTLA4 expression in high- and low-risk group.

The association between the risk score and expression of three immune checkpoint genes has been investigated in TARGET cohort (Figure 7). Our findings indicate that, in the high-risk group, PD-L1, PD-1, and CTLA4 were upregulated, which positively correlated with the high-risk score.

## 4. Discussion

AML is a malignant haematological disorder, with a rise in prevalence with age, and 70% of patients also die from the disease diagnosed [26]. Immune therapy advances have been met with many challenges for children and adults with AML, including lack of identified tumor-specific antiquities, inter- and intrapatient disease heterogeneity [27], as well as greater understanding of microenvironmental factors impeding the therapeutic effectiveness of immunosuppressive bone marrows [28]. Until now, checkpoint inhibitors in the pediatric leukemia community have been minimally examined. The latest opened Phase I study of Nivolumab with 5-azacytidine in children with multiple relapsed/refractory AML is examining the protection and tolerability, along with the assessment of the recommended Phase 2 dosage [29]. In the high-risk group, the expression levels of PD1, PD-L1, and CTLA4 were significantly increased, suggesting that when the AML patients are assigned to the high-risk group, there may be strong clinical indications for the use of immune checkpoint inhibitor treatment.

However, there are also not adequate immune checkpoint biomarkers and prognostic models for the survival of pediatric AML patients. The goal of this research was to develop an effective signature of prognosis for AML children and estimate the survival of AML children. Two datasets reported 128 common immune checkpoint-related genes. Immune checkpoint-related prognostic genes, which subsequently were analyzed in TARGET dataset using multivariate regression analysis, were screened using univariate Cox regression and LASSO algorithms in the TARGET dataset. Finally, a new three-gene model was developed and validated in GEO dataset, successively categorizing patients into groups with low risk and high risk with distinct OS, where there were considerably lower prognostic patterns than in high-risk group. The utility of the current signature suggests a good predictive potential. The 3-gene signature also shows that the survival of AML is an individual prediction. In addition, an immune reaction and immunotherapeutic reaction are more likely to occur in the high-risk group. Consequently, this gene-prognostic signature correlated with immune checkpoint is reliable, strong, and interpretable. In certain malignancies, tumor-infiltrating immune cells have a strong predictable tumor development and patient survival. The three genes were shown to be correlated to different immune cells.

Three risk genes have been found (STAT1, BATF, and EML4). STAT1 is a crucial IFN signaling portion. Most data suggest that activated STAT1 plays a function in cancer cells as a tumor suppressor [30]. In several forms of human cancer, such as breast cancers, pleural mesothelioma, head and neck cancer, and lymphoma, aberrant activation STAT1 has been identified [31]. In the majority of trials, high expression of STAT1 leads to improved clinical outcomes, but contradictive data has been shown suggesting that the clinical findings of cancer patients with high expression of STAT1 and/or pSTAT1 are poorer as contrasted with low expression patients [31]. Recent research showed that the eIF4F-STAT1-PD-L1 axis in melanoma is associated with immune evasion [32]. It has proven to be mainly expressed in hematopoietic cells, particularly in B and T cells. BATF belongs to the transcription factors family of AP-1. T cell activity can be blocked by the PD-1, which can reverse the signal downstream of TCR and CD28 co-stimulation by the enhanced production of the BATF transcription factors [33]. EML4 is a protein linked to microtubules that improve the stability of the microtubules [34]. Human EML4 is phosphorylated with mitosis residues of serine/threonine. A large proportion (∼5 percent) of lung adenocarcinoma patients as well as breast and colorectal tumors are found in pathological fusion sections of this gene with portions of ALK gene that produces the EML4-ALK transcript [35, 36]. In this research, the GEO dataset contains 422 adult patients which was used as validation group. A recent comprehensive genomic characterization of pediatric AML from Children's Oncology group showed that, though similar to adults, pediatric AML also has low rate of overall somatic mutation burden, and the mutational profile is different. Unlike adult AML, DNMT3A mutations and mutations in TP53 were almost absent and mutations in IDH1 or IDH2 were rare in pediatric patients [37]. In our research, the three genes involved in the model (STAT1, BATF, and EML4) have not been reported to be heterogeneous between adults and children. This may be part of the commonality between adults and children.

As far as we know, this is the first research to create a prognostic signature in pediatric AML dependent on an immune checkpoint. Our analysis, however, had some restrictions. Any additional intrinsic variables such as family background, gene mutation phenotypes, and the primary treatment process, which could have had an influence that may have had a certain influence on the outcome, were not feasible in this analysis. In other independent future research and operational tests on established genes, more confirmation of the utility of the signature is also required. In addition, more analytical capacity is expected in more clinical trials of greater sample sizes. So, before the effects can be translated to clinical practice, there is already a lot to be done.

## Figures and Tables

**Table 1:** Clinical data from TARGET and GEO datasets of AML patients.

Variables	Subgroups	TARGET (*n* = 156)	GSE37642 (*n* = 422)
Age			
	<14 years	103	—
	≥14 years	53	—
Gender			
	Male	91	—
	Female	65	—
Vital status			
	Alive	94	109
	Dead	62	308
	Unknown	0	5
WBC at diagnosis (10^6^/L)			
	≥50	77	—
	<50	79	—
Bone marrow leukemic blast percentage			
	≥90	32	—
	<90	120	—
	Unknown	4	—
CNS disease			
	No	146	—
	Yes	10	—

**Table 2:** Multivariate analysis of AML patients' OS identified independently prognostic factors in TARGET cohort.

	Multivariate analysis		
	HR	95%CI	*p* value
Gender	0.601	0.367–0.984	0.0432
WBC at diagnosis	1.001	0.585–1.711	0.9986
Bone marrow leukemic blast percentage	1.082	0.615–1.904	0.7829
CNS disease	1.774	0.693–4.541	0.2315
Age at diagnosis	1.044	1.001–1.089	0.0421
Risk score	1.914	1.366–2.682	0.0001

## Data Availability

The generated and analyzed datasets of the current research are available in TARGET database (https://ocg.cancer.gov/programs/target) and GEO database (https://www.ncbi.nlm.nih.gov/gds).

## Supplementary Materials

Supplementary Materials Figure S1. (a) PPI interactions among 128 immune checkpoint-related genes. (B) Word cloud for the 128 immune checkpoint-related genes. The size and color of each gene in word cloud correspond to the number of nodes connected with other genes. Table S1. A total of 282 specific candidate genes associated with immune checkpoint.
