# Supplementary material for: An Immune Checkpoint-Related Gene Signature for Predicting Survival of Pediatric Acute Myeloid Leukemia
Source: J Oncol. 2021 Apr 19;2021:5550116. doi: 10.1155/2021/5550116 (PMC8079183; doi:10.1155/2021/5550116)
Supplement: Supplementary Materials — Figure S1. (a) PPI interactions among 128 immune checkpoint-related genes. (B) Word cloud for the 128 immune checkpoint-related genes. The size and color of each gene in word cloud correspond to the number of nodes connected with other genes. Table S1. A total of 282 specific candidate genes associated with immune checkpoint. [file 5550116.f1.zip › 5550116.f1/FigureS1.pdf]

A.

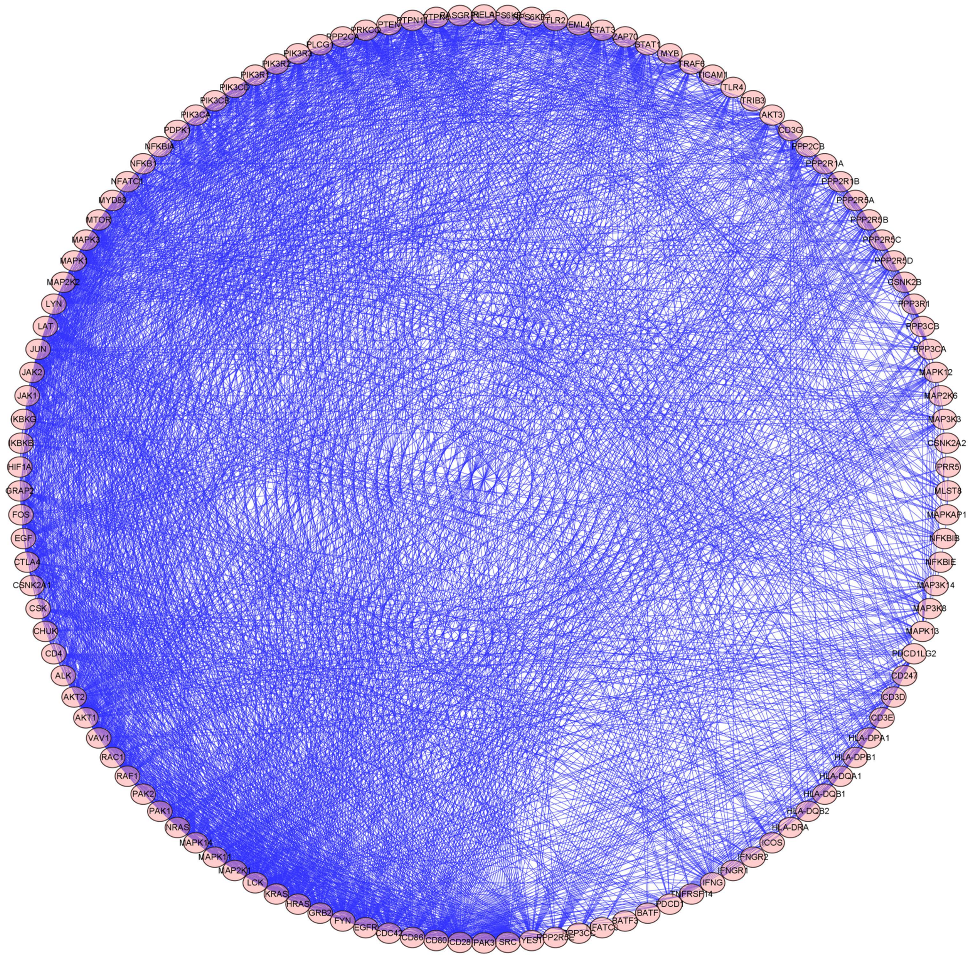

B.

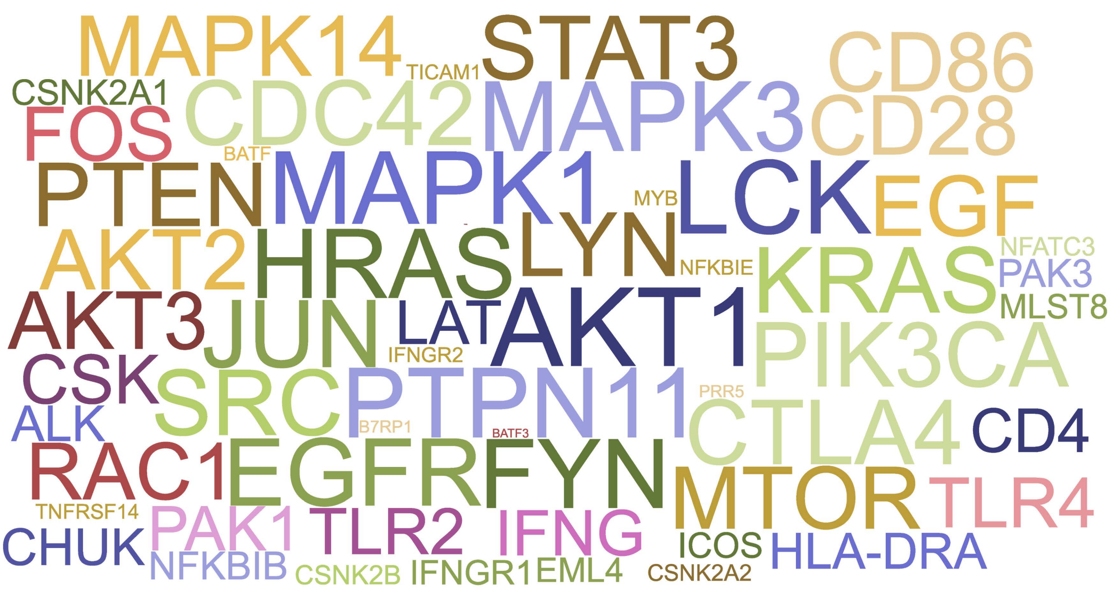
MAPK14
TICAM1
STAT3
CD86
CSNK2A1
FOS
CDC42
MAPK3
CD28
BATF
PTEN
MAPK1
MYB
LCK
EGF
AKT2
HRAS
LYN
NFKBIE
NFATC3
KRAS
PAK3
MLST8
AKT3
JUN
LAT
AKT1
IFNGR2
PIK3CA
CSK
SRC
PTPN11
PRR5
ALK
B7RP1
BATF3
CTLA4
CD4
RAC1
EGFR
FYN
MTOR
TLR4
TNFRSF14
PAK1
TLR2
IFNG
ICOS
CHUK
NFKBIB
CSNK2B
IFNGR1
EML4
CSNK2A2
HLA-DRA
