# Supplementary material for: An Immune Checkpoint-Related Gene Signature for Predicting Survival of Pediatric Acute Myeloid Leukemia
Source: J Oncol. 2021 Apr 19;2021:5550116. doi: 10.1155/2021/5550116 (PMC8079183; doi:10.1155/2021/5550116)
Supplement: Supplementary Materials — Figure S1. (a) PPI interactions among 128 immune checkpoint-related genes. (B) Word cloud for the 128 immune checkpoint-related genes. The size and color of each gene in word cloud correspond to the number of nodes connected with other genes. Table S1. A total of 282 specific candidate genes associated with immune checkpoint. [file 5550116.f1.zip › 5550116.f1/TableS1 (1).docx]

Table S1. A total of 282 unique candidate genes related to immune checkpoint from KEGG and Reactome.

|  |
| --- |
| AAM |
| ACS |
| ACUN |
| ACYG |
| AJU |
| AKT1 |
| AKT2 |
| AKT3 |
| ALK |
| AMEX |
| AMJ |
| AML |
| AOCE |
| APLA |
| ASN |
| BACU |
| BATF |
| BATF2 |
| BATF3 |
| BBUB |
| ZAP70 |
| PPP2R1B |
| SLAL |
| RAC1 |
| RTP |
| TLR4 |
| PPP2R5A |
| PPS |
| RRO |
| PDCD1LG2 |
| PDPK1 |
| PHI |
| PHYP |
| PIK3CA |
| PIK3CB |
| PIK3CD |
| PIK3R1 |
| PIK3R2 |
| PIK3R3 |
| PKI |
| PLCG1 |
| PMAJ |
| PMUA |
| PMUR |
| PON |
| POV |
| PPAD |
| PPP2CA |
| PPP2CB |
| PPP2R1A |
| TRAV29DV5 |
| TICAM2 |
| RPS6KB2 |
| SHR |
| TIRAP |
| PPP3R2 |
| PTR |
| NLE |
| NMEL |
| NNI |
| NRAS |
| OAA |
| OAS |
| OCU |
| OLA |
| ONL |
| OOR |
| ORO |
| OTW |
| PADL |
| PAK1 |
| PAK2 |
| PAK3 |
| PALE |
| PBI |
| PCAD |
| PCW |
| PDCD1 |
| TRBV12-3 |
| TICAM1 |
| RPS6KB1 |
| SGH |
| TRAV8-4 |
| PPP3R1 |
| PTPN6 |
| BIU |
| BOM |
| BTA |
| BTLA |
| CCAE |
| CCAN |
| CCAR |
| CCW |
| CD247 |
| CD274 |
| CD28 |
| CD3D |
| CD3E |
| CD3G |
| CD4 |
| CD80 |
| CD86 |
| CDC42 |
| CDK |
| CFA |
| TRAV19 |
| VAV1 |
| SRC |
| RAF1 |
| SALP |
| TLR9 |
| PPP2R5B |
| PRET |
| TRBC1 |
| DRO |
| EAI |
| ECB |
| EEE |
| EGF |
| EGFR |
| EGZ |
| ELK |
| ELS |
| EML4 |
| EPZ |
| ETL |
| FAB |
| FCA |
| FCH |
| FOS |
| FPG |
| FYN |
| GFR |
| GGA |
| VVP |
| UAH |
| SSC |
| RAY |
| SASA |
| TNFRSF14 |
| PPP2R5D |
| PRR5 |
| ICOS |
| ICOSLG |
| IFNG |
| IFNGR1 |
| IFNGR2 |
| IKBKB |
| IKBKG |
| IPU |
| JAK1 |
| JAK2 |
| JUN |
| KMR |
| KRAS |
| LAT |
| LAV |
| LCF |
| LCK |
| LCM |
| LSR |
| LVE |
| XLA |
| TSR |
| STAT3 |
| RELA |
| SCAN |
| TRAF6 |
| PPP3CA |
| PTEN |
| MGP |
| MJV |
| MLST8 |
| MMU |
| MNA |
| MPAH |
| MTOR |
| MUN |
| MYB |
| MYD |
| MYD88 |
| MZE |
| NFATC1 |
| NFATC2 |
| NFATC3 |
| NFKB1 |
| NFKBIA |
| NFKBIB |
| NFKBIE |
| NGI |
| XTR |
| TRBV7-9 |
| THEM4 |
| RNO |
| SFM |
| PVT |
| PPP3CC |
| PTPN11 |
| CFR |
| CGE |
| CHUK |
| CHX |
| CJC |
| CJO |
| CLV |
| CMK |
| CMY |
| CPIC |
| CSAB |
| CSK |
| CSNK2A1 |
| CSNK2A2 |
| CSNK2A3 |
| CSNK2B |
| CTLA4 |
| CVG |
| DLE |
| DRE |
| YES1 |
| UMR |
| SRX |
| RASGRP1 |
| SANH |
| TMU |
| PPP2R5C |
| PRKCQ |
| GGO |
| GJA |
| GRAP2 |
| GRB2 |
| HAI |
| HGL |
| HIF1A |
| HLA-DPA1 |
| HLA-DPB1 |
| HLA-DQA1 |
| HLA-DQA2 |
| HLA-DQB1 |
| HLA-DQB2 |
| HLA-DRA |
| HLA-DRB1 |
| HLA-DRB3 |
| HLA-DRB4 |
| HLA-DRB5 |
| HRAS |
| HSA |
| XCO |
| TUP |
| STAT1 |
| RBB |
| SBQ |
| TRAC |
| PPP2R5E |
| PSS |
| LYN |
| MALB |
| MAP2K1 |
| MAP2K2 |
| MAP2K3 |
| MAP2K6 |
| MAP3K14 |
| MAP3K3 |
| MAP3K8 |
| MAPK1 |
| MAPK11 |
| MAPK12 |
| MAPK13 |
| MAPK14 |
| MAPK3 |
| MAPKAP1 |
| MCAL |
| MCC |
| MCF |
| MDO |
| XMA |
| TRIB3 |
| TGU |
| RICTOR |
| SDU |
| TLR2 |
| PPP3CB |
| PTG |
